# First Report of Sylvatic DENV-2-Associated Dengue Hemorrhagic Fever in West Africa

**DOI:** 10.1371/journal.pntd.0001251

**Published:** 2011-08-02

**Authors:** Leticia Franco, Gustavo Palacios, José Antonio Martinez, Ana Vázquez, Nazir Savji, Fernando De Ory, María Paz Sanchez-Seco, Dolores Martín, W. Ian Lipkin, Antonio Tenorio

**Affiliations:** 1 National Center for Microbiology, Instituto de Salud Carlos III, Majadahonda, Madrid, Spain; 2 Center for Infection and Immunity, Mailman School of Public Health, Columbia University, New York, New York, United States of America; 3 Hospital Infanta Cristina, Parla, Madrid, Spain; 4 Laboratorio Br Salud, San Sebastián de los Reyes, Madrid, Spain; Centers for Disease Control and Prevention, United States of America

## Abstract

Dengue virus (DENV) circulates in human and sylvatic cycles. Sylvatic strains are both ecologically and evolutionarily distinct from endemic viruses. Although sylvatic dengue cycles occur in West African countries and Malaysia, only a few cases of mild human disease caused by sylvatic strains and one single case of dengue hemorrhagic fever in Malaysia have been reported. Here we report a case of dengue hemorrhagic fever (DHF) with thrombocytopenia (13000/µl), a raised hematocrit (32% above baseline) and mucosal bleeding in a 27-year-old male returning to Spain in November 2009 after visiting his home country Guinea Bissau. Sylvatic DENV-2 West African lineage was isolated from blood and sera. This is the first case of DHF associated with sylvatic DENV-2 in Africa and the second case worldwide of DHF caused by a sylvatic strain.

## Author Summary

Dengue viruses are mosquito borne pathogens that cause up to 100 million infections annually. Dengue viruses circulate in either humans or non-human primates in two separate cycles: human and sylvatic. All four different serotypes infect humans causing frequent epidemics and severe disease, including hemorrhagic fever. Until a few years ago, viruses circulating in the sylvatic cycle had been associated with asymptomatic to mild disease only in humans. For example, retrospective analysis of human serum samples collected in Nigeria from the 1960s proved that sylvatic dengue was able to cause an outbreak of dengue fever in human population. Recently a case of DHF in Asia was linked to sylvatic DENV-2 (strain DKD811) infection. Here, we report the first case of DHF associated with sylvatic DENV-2 infection in Africa caused by a complete different lineage of the virus. All these events underscore the need to be vigilant about the re-emergence of DHF from sylvatic cycles.

## Introduction

Dengue viruses (DENV) are the most widely distributed arboviruses in the world. The four distinct serotypes belong to the family *Flaviviridae* and are some of the most important vector-borne pathogens of humans [1]. DENV circulation occurs in two cycles: an endemic/epidemic cycle between humans and peridomestic mosquitoes, *Aedes aegypti* and *Ae. albopictus*, and a sylvatic enzootic cycle between non-human primates and several arboreal *Aedes* species. In Asia, circulation of sylvatic DENV-1, -2 and -4 has been detected in *Ae. niveus* mosquitoes and/or sentinel monkeys [2]. Although sylvatic DENV-3 strains have not been isolated to date, they are believed to circulate in Malaysia based on seroconversion of sentinel monkeys [3], [4]. In Africa, only sylvatic DENV-2 has been reported as associated with *Ae. luteocephalus, Ae. taylori, Ae. furcifer* and other forest gallery mosquitoes [5]. Sylvatic DENV strains are ecologically and evolutionary different from endemic strains [6] and the Asian (Malaysia) and West African lineages of sylvatic DENV-2 are clearly divergent [7].

**Figure 1:**
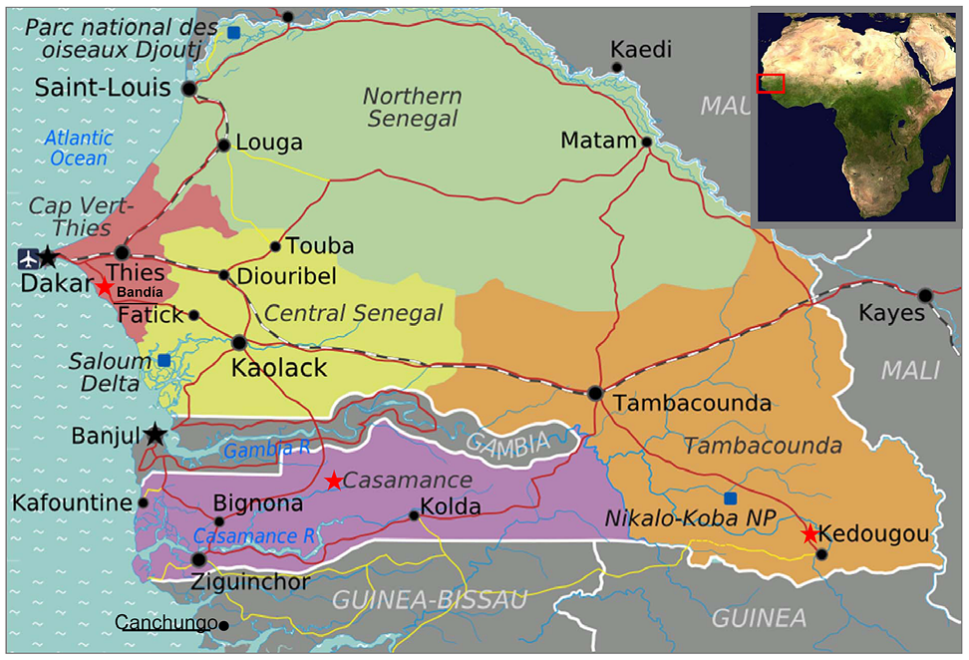
Geographical context. Map of Senegal showing regions and boundaries' with: The Gambia, Guinea Bissau and Mali. Areas with report of sylvatic dengue activity are signaling by red stars. Canchungo town is underlined. The patient travelled from Canchungo to Dakar through Casamance, Central Senegal and Capt Vert-Thies regions. Figure was created using maps from http://www.worldofmaps.net (The map of Senegal is licensed under the Creative Commons Attribution-Share Alike 3.0 and the map of Africa is created by the NASA and is of public domain).

Since the 1970s in Africa, sylvatic DENV-2 has been detected primarily in mosquitoes in south eastern Senegal (Kedougou region), but also in Guinea, Ivory Coast and Burkina Faso [5], [7], [8], [9] with sporadic isolations from humans and monkeys [10], [11], [12], [13]. Epizootic DENV-2 was first described in the region of Kedougou in Senegal in 1974, and successively in 1980-82, 1989-90 and 1999-2000, with a periodicity of approximately 8 to 9 years [5]. The first isolation of sylvatic DENV-2 from a human was reported in 1970 in Bandia, Senegal (sixty kilometers east of Dakar) from a young girl [14], [15]. Subsequently, DENV-2 was isolated in 1983 from a French expatriate in south western Senegal in the Casamance region [13] bordering the Gambia and Guinea Bissau (**Figure 1**) and in 1990 in south eastern Senegal [10].

The first documented outbreak of sylvatic DENV in humans was identified in a retrospective study on samples collected in Ibadan, Nigeria from August 1964 to December 1968 [16]. Thirty-two strains of DENV were recovered of which 14 were DENV-2 [12]. Three complete genomes from samples taken in 1966 were obtained and analyzed and found to be West African sylvatic DENV-2 [16]. These findings suggested that DENV-2 was enzootic in West Africa at least 40 years ago with a limited spillover into humans. More recently, in Malaysia in 2005, a DENV-1 detected from a febrile patient clustered with the ancestral sylvatic DENV-1 isolated from a sentinel monkey in 1972 (strain P72_1244)[17]. However, recent evidence based on complete genome phylogenetic analysis suggests this strain falls into the human dengue diversity of endemic DENV-1 [18].

The epidemic and pathogenic potential of sylvatic DENV strains remains controversial. Some authors argue that sylvatic viruses do not pose a threat to public health [19]. In 2008, two events were linked to sylvatic dengue virus activity in humans in West Africa and Asia. Two imported dengue fever cases were reported in France in travelers returning from West Africa, Mali and Senegal [20]; concurrently a dengue fever outbreak occurred in a town 450 km from Bamako, Mali [21]. Genetic analysis of isolates from both sites revealed 99.6% sequence identity; phylogenetic analysis revealed that all sequences clustered with the West African sylvatic DENV-2 [21]. Almost simultaneously, the first documented case of dengue hemorrhagic fever (DHF) involving sylvatic DENV was observed in Malaysia [22]. A student returning from a trip to peninsular Malaysia was diagnosed with grade II DHF associated with sylvatic DENV-2. This 2008 Malaysian strain sequence [22] clustered with the sequences of Asian sylvatic dengue strains recovered from monkeys in the 1970s.

Here, we report the first African case of DHF associated with sylvatic DENV-2 of a West African lineage.

## Materials and Methods

### Case introduction description

A previously healthy young male native to Guinea Bissau, but living in Spain, was admitted to Emergency Services at the Infanta Cristina Hospital, Madrid, Spain. The patient had returned to Madrid after a 3-month trip to his home country. When admitted, the patient presented with fever, headache, conjunctival hyperemia, and musculoskeletal pain. He did not have nausea, vomiting, voiding symptoms, or skin lesions. Cardiopulmonary examination yielded no findings. The patient was normotensive with good baseline oxygen saturation. He had not received any type of prophylaxis for malaria or other agents before the trip. During hospitalization, blood samples were obtained for virological and serological testing at the National Centre of Microbiology (CNM), Instituto de Salud Carlos III. Dengue case classification and management was performed according to WHO criteria [23] and subsequently adjusted to the new WHO/TDR guidelines [24].

### Ethics statement

The institutional review board at the Instituto Carlos III approved this study under the PI08/0834 identification. Through this protocol, the patient's identity was un-linked from the sample, test result, and Genbank accession number. EEB-17 is an arbitrary identifier assigned to the viral culture sample used to sequence the genome and thus cannot be linked with the patient's identity.

### Virological and serological testing

Whole blood and serum obtained at 5 and 6 days post onset respectively were processed for virological and serological tests (**Table 1**). Viral isolation was attempted from serum and blood samples inoculated into C6/36 mosquito cells. Five days post infection; cells were harvested after observation of evident cytopathic effect. This viral isolate was assigned the strain name EEB-17. DENV-2 was identified by indirect immunofluorescence assay using the commercial monoclonal antibody 8702 (clone 3H5-1), (Chemicon, Temecula, CA, USA). Viral supernatant was inactivated in lysis buffer (AVL, Qiagen, Valencia, CA, USA) for nucleic acid extraction and PCR amplification.

**Table 1:** Analytical and virological findings.

	Days post onset (date)									
	4^a^(11/09)	5^b^(11/10)	6^c^(11/11)	7(11/12)	8(11/13)	9(11/14)	10(11/15)	11(11/16)	12^d^(11/17)	24^e^(11/29)
Platelet (µL)	25000	19000	13000	14000	16000	32000	51000	81000	103000	211000
White cells (µL)	3700	3900	11600	14500	7200	5000	6000	5680	5600	3460
Hemoglobin (g/dL)	16.9	17.3	16.1	14.5	14	13.5	14	14.5	15.1	13.6
Hematocrit (%)	51.2	51.8	48.2	43.5	42	40.4	41.9	42.4	45.3	39.1
Hemoconcentration (%)	30.9^f^	32.5^f^	23.3^f^	11.3	7.4	3.3	7.1	8.4	16	-
Ac protrombin (%)	78	81.6	104	98.6	104			102		95
Crp (mg/dL)	0.9	0.7	0.6	0.7	0.6			0.7	0.8	0.7
GLU (mg/dl)	85	99	75	67	67			78	77	85
GPT (U/L)	52	80	133	207	162			156	160	64
GOT (U/L)	134	188	377	620	429			208	185	50
GGT (U/L)								230		
LDH (U/L)			1249	1270	747			523	503	246
Tot Bilirub. (mg/dL)	0.5	0.4	0.4	0.2	0.2			0.3	0.5	0.2
Alk. Phosp. (U//L)	105	99		124	110			96		106
C Reactive Protein (U/L)	9.8	7.4	9.2	10.3	<5			0.1		0.1
MAC-ELISA(OD)			40.9							
ELISA IgG (OD)			58.8							
IgG Avidity ELISA			High avidity							
NS1 Antigen (OD)			10.8							
RT-PCR (E/NS1)		Positive	Positive							
Virus isolation		DENV-2	DENV-2							
RT-PCR(NS5)^g^		Positive	Positive							

OD: adjusted optical densities.

a Day of admission.

b Day whole blood was obtained.

c Day serum was obtained.

d Day of discharge.

e Day of re-evaluation.

f >20% of hemoconcentration.

g in viral isolates.

IgM and IgG antibodies were determined by capture and indirect-ELISA, respectively (Panbio, QLD, Australia). As previously described [25], IgM reactivity was confirmed by the performance of a background assay (e.g. parallel assessment with presence or absence of antigen). NS1 antigenemia was determined by using PLATELIA dengue NS1 capture Antigen ELISA (BioRad, Marnes la Coquette, France). Finally, to differentiate primary from secondary infection an avidity IgG test was done [25].

Sera, blood and viral supernatants, inactivated with AVL/carrier RNA solution Buffer (Qiagen), were RNA extracted with the QIACube (Qiagen) extractor. RT-PCR and product sequencing were performed as previously described [26]. A different RT-PCR technique that amplifies 1019 bp of the NS5 flaviviral gene [27] was used as a confirmatory test in the supernatants of the viral isolates. RT-PCR products were detected by gel electrophoresis and purified by the QIAquick PCR purification kit (Qiagen) and sequenced with ABI prism Big Dye terminator cycle sequencer v3.1 ready reaction (Applied Biosystem, Foster City, CA, USA) and analyzed on the ABI prism 377 DNA Analyzer (Applied Biosystem).

To sequence the genome, primers were designed to produce amplicons of 1000 bps with 500 bp overlaps based on Dengue virus type 2 isolate Dak Ar D75505, complete genome (EF457904), which was, based on similarity analysis of the consensus NS5 PCR amplicon, the closest relative to our strain (**Table S1**). A total of 31 amplicons, approximately 900 bps in length were used to produce 62 chromatograms. This provided greater than four times coverage of the complete genome with 400–450 bp overlap amongst one another. The accession number of the complete genome of EEB-17 is JF260983, which is 10,176 bps long and includes the complete open reading frame of the polyprotein.

Conventional PCRs were performed with HotStar polymerase (Qiagen) on PTC-200 thermocyclers (Bio-Rad, Hercules, CA, USA): an enzyme activation step of 5 min at 95 °C was followed by 45 cycles of denaturation at 95°C for 1 min, annealing at 55°C for 1 min, and extension at 72°C for 1 min. Amplification products were size fractionated by electrophoresis in 1% agarose gels, purified (MinElute, Qiagen), and directly sequenced in both directions with ABI PRISM Big Dye Terminator 1.1 Cycle Sequencing kits on ABI PRISM 3700 DNA Analyzers (Applied Biosystems).

### Phylogenetic analysis

To determine the evolutionary history of isolate EEB-17, we performed a phylogenetic analysis on all available complete genome sequences of DENV-2 (total alignment length of 10,173 nt; 906 sequences, Oct 15, 2010). First, we performed a clustering analysis to group available sequences at 97; 95 and 90% similarity using the CD-HIT clustering program at http://weizhong-lab.ucsd.edu/cdhit_suite/cgi-bin/index.cgi. This step allowed us to exclude similar sequences, identify clusters corresponding with known lineages and identify the more divergent members of each cluster for proper phylogenetic analysis.

After identification of clusters in correspondence with the accepted groupings of DENV-2 genotypes, we identified representative sequences from each separate cluster to perform deep phylogenetic analysis. Specifically, we inferred a Maximum Clade Credibility (MCC) tree using the Bayesian Markov Chain Monte Carlo (MCMC) method available in the Beast package [28], thereby incorporating information on virus sampling time. This analysis utilized a strict molecular clock and a GTR+Γ model of nucleotide substitution for each codon position, although very similar results were obtained using other methods (data available upon request). The analysis used a Bayesian skyline model as a coalescent prior as was recently reported in a similar study [22]. All chains were run until convergence for all parameters with 10% removed as burn-in.

The analysis also allowed us to estimate divergence times for each node in the DENV-2 genotypes. The degree of uncertainty in each parameter estimate is provided by the 95% highest posterior density (HPD) values, while posterior probability values provide an assessment of the degree of support for each node on the tree.

## Results

### Clinical and epidemiological data

On November 9, 2009, a 27-year-old man travelling from Guinea Bissau was admitted to the Hospital Infanta Cristina in Parla, Madrid, after returning from his home country. He had spent 3 months in the city of Canchungo, in the northwestern coast of Guinea Bissau. The patient had initiated his return trip from Canchungo to Dakar, Senegal on October 28, by car, through the Casamance region in south-western Senegal (**Figure 1**). He stayed in Dakar for approximately a week before travelling by air to Spain.

On November 6, the patient arrived in Madrid. He reported feeling ill during the flight. At admission, he presented with fever (38.6 °C), conjunctival hyperemia, headache and musculoskeletal pain but no nausea or vomiting, voiding symptoms, or skin lesions. During his intake history, the patient reported insect bites during his recent travel to Dakar.

Laboratory investigation demonstrated leukopenia with marked left shift, and significant thrombocytopenia (13000/µl). No alteration of clotting time was observed. Renal function was normal. Liver function impairment was demonstrated by elevated lactate dehydrogenase (LDH), alanine transminase (ALT) and aspartate transaminase (AST). Normal values were obtained for total bilirubin (TBIL) and direct bilirubin, gamma glutamyl transpeptidase (GGT), amylase and lipase demonstrating normal cholestasis data (**Table 1**). Radiological studies of the chest and abdomen were normal.

After admission to internal medicine service for fever and thrombocytopenia, the patient experienced epigastric pain, without hemodynamic compromise. Follow-up analytical testing showed a progressive decline in platelet numbers, elevated liver enzymes and hemoglobin with normal bilirubin. Malaria, parasites in blood, feces and urine, as well as HIV, HAV, HCV and HBV infections were ruled out. Considering the epidemiological context and data concerning the fever, thrombocytopenia, abdominal pain, elevation of liver enzymes and negative results for malaria, dengue virus infection was suspected. One day after admission, samples were taken and sent to the CNM for dengue PCR and the next day for serology.

During an abdominal ultrasound no evidence of injury to the liver or spleen was observed. Gallbladder and bile ducts were also normal. On the other hand, the presence of free liquid in the sub-hepatic area and pouch of Douglas were remarkable. During hospitalization, the patient also experienced episodes of self-limited epistaxis, bleeding from venipuncture sites, episodes of hemoptoic sputum without respiratory or hemodynamic impact, and increased hyperemia with self-limited subconjunctival hemorrhage. Furthermore, the hemoconcentration observed on the 5th day after onset was very high having risen more than 32% above baseline.

While admitted, the patient was only treated with supportive care (hydration and non-inflammatory analgesia), without requiring platelet transfusion. After four days hospitalized, the number of platelets, liver function and general clinical symptoms started to show improvement; no new episodes of bleeding or abdominal pain were observed. Eight days after admission the patient was discharged.

According to the original WHO definition of DHF [23], the case met the four criteria for classification as grade II DHF (fever, thrombocytopenia, hemorrhage and plasma leakage). However, according to the newly proposed WHO definition [24], the case would be classified as “dengue with alarm signs.”

By day 22, the patient was clinically asymptomatic. All analytical parameters were normal with a slight increase of AST and ALT (**Table 1**).

### Virological and serological data

DENV-2 was found in blood and sera in the samples taken 4 and 5 days post onset of fever by routine consensus PCR over the E/NS1 junction [26]. PCR amplification in the NS5 was also performed to confirm the initial results (PCR E/NS1 and IFI). BLAST analysis of the sequence obtained from the PCR products gave a similarity index of 97% with sylvatic West African lineage DENV-2 (Accession number EF457904). Both IgM and IgG were positive in the sera taken at day 6 after onset. The quantitative results obtained (40.9 for IgM and 58.8 for IgG) suggested a secondary infection. The avidity assay characterized the IgG response as high avidity. NS1 antigen was detected in the sample at high concentration.

### Phylogenetic analysis

**Figure 2:**
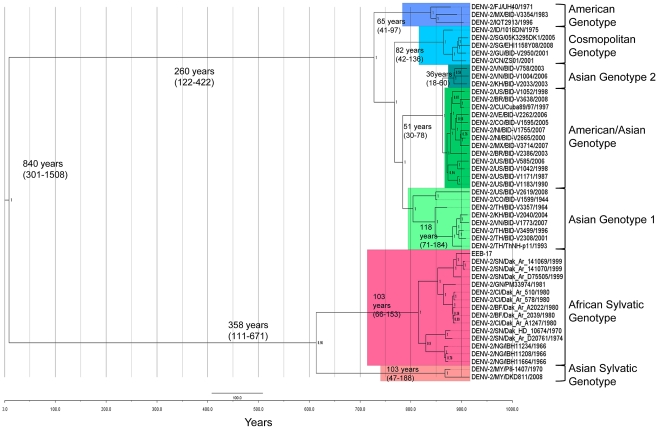
Maximum Clade Credibility (MCC) tree of 906 complete genomes of DENV-2. The six major genotypes are highlighted with different colors. Sylvatic genotypes from West Africa and Malaysia are colored in red and pink, respectively. The 95% HPD values for most recent common ancestor are labeled beside the node. Strains are labeled as follow: serotype/country/strain name/year. EEB-17 accession number: JF260983. Genbank accession numbers of sequences presented in this figure are listed in Text S1.

Phylogenetic analysis from the E/NS1 and NS5 regions revealed that the sequences fell into DENV 2 sylvatic genotype group, within the African isolates (data not shown). Complete genome phylogenetic analysis confirmed that EEB-17 fell within the West African sylvatic lineage of DENV-2 and was most closely related to isolates DakAr141069 and DakAr141070 [7] (**Figure 2**). Both isolates were detected in 1999 in *Ae. luteocephalus* mosquitoes from Kedougou region (South-eastern Senegal) [5]. Interestingly, sylvatic strains of African origin showed a clustering by year of isolation. This phylogenetic history was supported by high posterior probability values (1.0). Molecular clock analysis suggests that the African sylvatic lineage shares a common ancestor approximately 103 years ago (95% HPD of 66–153 years). Thus, DENV-2 has likely been circulating in non-human primates in Africa for at least this long. Our analysis also predicts the Time to the Most Recent Common Ancestor (TMRCA) for each of the human genotypes demonstrating that the divergence of the Asian and African lineages of sylvatic DENV-2 is comparable to the observed difference among human lineages. The mean evolutionary rates estimated ranged from 3.7×10^−4^ substitutions per site per year (95% HPD, 1.8×10^−4^ to 5.9×10^−4^).

## Discussion

Although sylvatic dengue cycles occur in West African countries and Malaysia, only a few cases of human disease caused by sylvatic strains have been reported [5]. Given that infection with sylvatic DENV may result in clinical presentation indistinguishable from that presented due to infection with strains from the human transmission cycle, it is possible that cases of sylvatic dengue are underreported. Nevertheless, after 30 years of silence, sylvatic dengue has re-emerged causing severe disease in a young man [22]. Although silent transmission during those 30 years is possible (human or sylvatic), no reports suggest it has occurred. We describe here an imported DHF case caused by a sylvatic strain in a healthy man returning to Madrid from Guinea Bissau through Senegal. This is the first report of DHF caused by a sylvatic DENV-2 West African lineage virus. The patient had dengue fever with alarming symptoms, such as haemorrhagic manifestations (mucosal bleeding, hematocrit 32% above baseline), thrombocytopenia and fluid accumulation in the abdominal cavity associated with abdominal pain. Under the old WHO definition, the patient classifies as grade II DHF with a risk of developing into grade III [23]. As indicated by the IgG avidity test, the patient showed high avidity antibodies suggesting a secondary flavivirus infection. Secondary infection with sylvatic DENV may have resulted after a primary infection with another dengue serotype, considered a risk factor for development of DHF[29]; after a primary infection with another flavivirus, since such viruses circulate throughout Guinea Bissau and neighbouring nations [30]; or after the development of immunity resulted by YFV vaccination. The IgM/IgG ratio or IgG avidity index has limitation to differentiate true dengue secondary infection in individuals with previous immunity against other flavivirus[25]. Evidence of previous infection with another DENV serotype might explain disease severity. Lack of evidence would suggest severe disease in a primary case of dengue caused by a sylvatic strain. Unfortunately, we cannot demonstrate previous infection with another DENV serotype. DENV is endemic in West Africa; and the predominant serotype with sustained circulation in the region is DENV-2 from sylvatic or epidemic lineage [31]. DENV-3 was recently detected in Ivory Cost [32] and it continues to expand throughout the region [20], [33]. In Dakar, Senegal, DENV-3 was detected in 2009 [20].

Based on the incubation period of dengue and previous knowledge describing the zone of circulation of sylvatic DENV-2 strains in Senegal, we posit that the infection was acquired during the overland trip from Canchungo, Guinea-Bissau to Dakar, Senegal, via Casamance, a region of documented sylvatic DENV activity [13]. The following points were considered to build that hypothesis: i) sylvatic DENV-2 has been frequently isolated in Senegal [5]; ii) our isolate EEB-17 is closely related to isolates obtained from *Ae. luteocephalus* captured in Senegal in the year 1999; iii) absence of sylvatic dengue circulation in Guinea-Bissau; iv) During October 2009, DENV-3 circulation was identified in Dakar [20], [34], the same time this DENV-2 case was identified. Moreover, outbreaks in Dakar and in the Kedougou region were reported [20]. In the Kedougou region, a sylvatic amplification cycle was detected with the isolation of sylvatic strains during surveillance activities by Institute Pasteur of Dakar (Dr. Amadou Sall, personal communication); and vi) Circulation of sylvatic DENV-2 would follow the same pattern of periodicity of the epizootics in the area with an interval cycle of approximately 8–9 years [5].

In addition, our complete genome phylogenetic analysis revealed that EEB-17 belongs to the West African lineage, along with isolates post-1980. According to the molecular clock, the African sylvatic lineage has shared a common ancestor approximately 103 years ago. These values are in agreement with previous reports [7], [22]. The mean evolutionary rates estimated were in complete concordance with the rates obtained by Vasilakis et al [7].

Introduction of DENV in Europe was observed in the summer of 2010. Autochthonous circulation of DENV was detected in France [35] and Croatia [36]. Mosquito surveillance in the countries on the Mediterranean basin demonstrates the presence of *Ae. albopictus*, and highlights the risk of initiation of a local transmission cycle in the presence of high vector densities. In that context, DENV strains could be introduced in naïve countries with the potential to develop limited or even extensive outbreaks, such as occurred with the introduction of Chikungunya virus in Italy in 2007 [37].

In summary, we described here the first case of DHF grade II caused by a sylvatic DENV-2 belonging to a West African lineage virus. The findings suggest that sylvatic strains of DENV might have a greater pathogenic potential than previously thought [19].

## Supporting Information

Table S1 List of primers used to amplify the complete genome of isolate EEB17.
(DOC)

Text S1 Genbank accession numbers of sequences presented in Figure 2.
(DOC)
