# Supplementary material for: First Report of Sylvatic DENV-2-Associated Dengue Hemorrhagic Fever in West Africa
Source: PLoS Negl Trop Dis. 2011 Aug 2;5(8):e1251. doi: 10.1371/journal.pntd.0001251 (PMC3149010; doi:10.1371/journal.pntd.0001251)
Supplement: Table S1 — List of primers used to amplify the complete genome of isolate EEB17. (DOC) [file pntd.0001251.s001.doc]

**Table S1: Primers used for amplification of the complete genome of isolate EEB17**

| **Primer** | **Sequence** |
| --- | --- |
| EF457904_1F | GTTAGTCTACGTGGACCGACAAAG |
| EF457904_2F | GTGTACCCTTATGGCCATGGATTT |
| EF457904_1R | TCCACGAAGTCTCGGTTTGATATG |
| EF457904_3F | TATCGTCTTAGAACACGGAAGCTG |
| EF457904_2R | ATTCCATCGTGATCGTGCCATAAT |
| EF457904_4F | TAATGAGATGGTCCTGCTGCAGAT |
| EF457904_3R | CCAAGTCCATGATCTCAAAGGGAA |
| EF457904_5F | AATCAACATAGAAGCAGAACCCCC |
| EF457904_4R | AGTCACAAAGATTCCACTTCCACA |
| EF457904_6F | ACAAATTTCAACCAGACTCCCCTT |
| EF457904_5R | GCTGACATCAACTTTGAATCACAAATC |
| EF457904_7F | ATGGGCTATTGGATAGAGAGCAGA |
| EF457904_6R | CCAATGAGTTCACCAGGTTCTCTT |
| EF457904_8F | TCTCACTAGGGATTCTGGGAATGG |
| EF457904_7R | TGCTGGAGTATCATGACATTTGGA |
| EF457904_9F | TATCACCACTCCTCTTGACTTCCT |
| EF457904_8R | CTGTGATTGGGATTGATACTGGGA |
| EF457904_10F | GTGCTGTGGGACGTTCCTTC |
| EF457904_9R | GAGACCCACGACTTTTCCTTTCTT |
| EF457904_11F | CCAAACAGAGAAAAGCGTAGAGGA |
| EF457904_10R | CTGGAGGAGTGGCTGTCATAAATA |
| EF457904_12F | ATGGAATTCCGGACATGAATGGAT |
| EF457904_11R | CCAGTGAGCACAGTCTTCATCATT |
| EF457904_13F | GAAGGTATCATCCCCAGCATGTTC |
| EF457904_12R | TATAGGCTCTTCCGCCCATTTCT |
| EF457904_14F | CTCTCAGTGAACTGCCAGAAACTC |
| EF457904_13R | CAGCATTGGCGTAATAAACGTAGT |
| EF457904_15F | TGTGTCACTAACAGCCATAGCAAA |
| EF457904_14R | TTTTCCTGGATTTCCTTCCCACAG |
| EF457904_15R | AAGAATACATCCACTCCACTCTGC |
| EF457904_16F | GAATCATCACCAAACCCCACAGTA |
| EF457904_17F | TCATCAATGGTAAACGGAGTGGTC |
| EF457904_16R | TGCCAAATTCTCCCAGTTTCTTCT |
| EF457904_18F | CTAGGTGCCCGCTTTTTAGAGTTT |
| EF457904_17R | TCTTGGGGTGTTAAGTGCAGTATG |
| EF457904_19F | CAAGAATGGCAATCAGTGGAGACG |
| EF457904_20F | AAACCAGGATGAACTGATAGGCAG |
| EF457904_18R | TATGGACCAAGTTGTCCTGCTAGT |
| EF457904_19R | CTAGTCCACTACACCATGCGTACA |
| EF457904_20R | CAACAGCACCATTCCATTTTCTGG |
