# Supplementary material for: First Report of Sylvatic DENV-2-Associated Dengue Hemorrhagic Fever in West Africa
Source: PLoS Negl Trop Dis. 2011 Aug 2;5(8):e1251. doi: 10.1371/journal.pntd.0001251 (PMC3149010; doi:10.1371/journal.pntd.0001251)
Supplement: Text S1 — Genbank accession numbers of sequences presented in Figure 2. (DOC) [file pntd.0001251.s002.doc]

**Supplementary Material S1: Genbank Accession numbers of sequences presented in Figure 2**

HM582100, GQ868588, AF100468, GQ398258, EU081177, GU370051, HM488257, EF051521, EU482780, EU482449, FJ639703, EU482560, GU131789, AY702037, FJ639822, FJ024474, FJ744743, FJ850066, GQ868516, FJ850078, EU529706, EU482554, EU482575, EU482586M GQ199899, EU854293, GQ868591, FJ639706, FJ410193, GQ868544, FJ744722, AF022437, EF105389, EF105390, EF457904, EF105378, EF105381, EF105380, EF105386, EF105382, EF105383, EF105384, EF105385, EU003591, EF105387, EF105388, EF105379, FJ467493
